# Synthesis of New Quinoline-Piperonal Hybrids as Potential Drugs against Alzheimer’s Disease

**DOI:** 10.3390/ijms20163944

**Published:** 2019-08-14

**Authors:** Juliana de Oliveira C. Brum, Denise Cristian F. Neto, Joyce Sobreiro F. D. de Almeida, Josélia Alencar Lima, Kamil Kuca, Tanos Celmar C. França, José D. Figueroa-Villar

**Affiliations:** 1 Medicinal Chemistry Group, Department of Chemistry, Military Institute of Engineering, Praça General Tibúrcio 80, 22290-270 Rio de Janeiro, RJ, Brazil; 2 Laboratory of Molecular Modeling Applied to the Chemical and Biological Defense (LMCBD), Military Institute of Engineering, Praça General Tibúrcio 80, 22290-270 Rio de Janeiro, Brazil; 3 Graduate Program in Adult Health, Federal University of Maranhão, Avenida dos Portugueses, 1966. Vila Bacanga, 65080-805 São Luís, MA, Brazil; 4 Department of Chemistry, Faculty of Science, University of Hradec Kralove, Rokitanskeho 62, 50003 Hradec Kralové, Czech Republic

**Keywords:** piperonal, quinolines, guanil-hydrazones, acetylcholinesterase, Alzheimer’s disease

## Abstract

Six quinoline-piperonal hybrids were synthesized and evaluated as potential drugs against Alzheimer’s disease (AD). Theoretical analysis of the pharmacokinetic and toxicological properties of the compounds suggest that they present good oral bio-availability and are also capable of penetrating the blood–brain barrier, qualifying as leads for new drugs against AD. Evaluation of their inhibitory capacity against acetyl- and butyrilcholinesterases (AChE and BChE) through Ellmann’s test showed that three compounds present promising results with one of them being capable of inhibiting both enzymes. Further docking studies of the six compounds synthesized helped to elucidate the main interactions that may be responsible for the inhibitory activities observed.

## 1. Introduction

Alzheimer’s disease (AD) is a progressive and neurodegenerative disease recognized today as the most common cause of dementia worldwide [1,2,3]. During the manifestation of this disease an atrophy of the Meynert nucleus basalis occurs in the brain, resulting in lowering levels of the neurotransmitter acetylcholine (ACh). This compromises the transmission of nerve impulses in the patient body, causing serious damages on his cognitive functions, characteristic of AD. The therapeutic approach to counteract this effect is raising the concentration of ACh through the inhibition of the enzymes acetyl- and butyrilcholinesterase (AChE and BChE), which are responsible for the hydrolysis of ACh in the synaptic clefts [4]. For this reason the cholinesterase inhibitors have been the first line drugs for treatment of light to moderate manifestations of AD. These drugs are the majority of the few drugs approved so far for the treatment of AD [5].

Donepezil, rivastigmine and galantamine are the cholinesterase inhibitors approved today for clinical use against AD. Donepezil and galantamine are selective inhibitors of AChE, while rivastigmine is a dual inhibitor of both AChE and BChE [6]. However, these drugs can cause side effects like nausea, vomiting and diarrhea, in addition to cardiovascular and neurologic effects.

**Figure 1:**
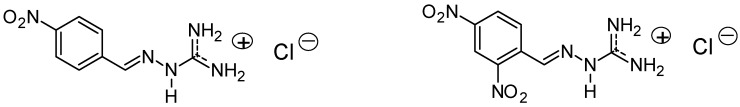
Structures of guanylhydrazones capable of inhibiting acetylcholinesterase (AChE) reported by Petronilho et al. [13].

Tacrine is also an effective cholinesterase inhibitor for the symptomatic treatment of AD. It is capable of avoiding the excessive degradation of ACh, increasing the cholinergic neurotransmission [7]. However, this drug is also capable of raising the serum levels of the enzyme alanine aminotransferase, provoking considerable hepatoxicity. For this reason the use of tacrine was discontinued in USA in 2013 [8]. Its quinolinic nucleus, however, presents a wide range of pharmacologic activities, being for this reason incorporated into a wide variety of compounds [9,10,11]. Considering the pharmacological potential of the quinolinic nucleus of tacrine we investigated in this work hybrid compounds, merging it with piperonal, and evaluated their cholinesterase activities. Our compounds contain the 3,4-dioxole bridge, known as a good acceptor of hydrogen bonds—a fact that may increase their capacity of inhibiting AChE [12]. We also evaluated the inclusion of a guanylhydrazone group in order to investigate the effects of this group inside AChE. This functional group is still little investigated in the literature as a cholinesterase inhibitor. Our group has pioneered this research in a previous work [13] where we report the results of the guanylhydrazones shown in Figure 1 as potential cholinesterase inhibitors. These compounds were evaluated against AChE through Ellman’s and NMR studies and presented inhibitory activity of AChE from *Electrophorus electricus* (EeAChE), confirming the guanylhydrazones as potential inhibitors of AChE. We believe that this activity is due to the presence of the cationic groups, able to interact with the peripheral anionic site (PAS) of AChE [13].

## 2. Results and Discussion

### 2.1. Synthesis of the Quinoline-Piperonal Hybrids

**Scheme 1:**
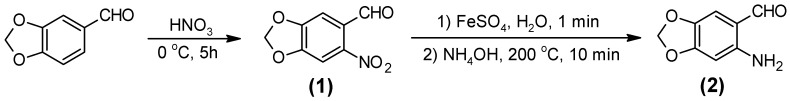
Synthesis of intermediates (**1**) and (**2**).

The synthetic route of this work started with the transformations of piperonal into intermediates 6-nitro-1,3-benzodioxole-5-carbaldehyde **(1)** and 6-amino-1,3-benzodioxole-5-carbaldehyde **(2)**, as shown in Scheme 1 [14,15,16]. 

**Scheme 2:**
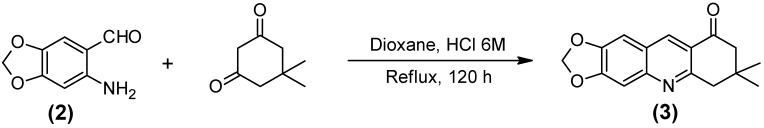
Synthesis of compound (**3**).

**Scheme 3:**
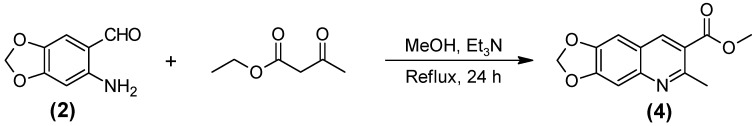
Synthesis of compound (**4**).

The hybrids piperonal-quinoline were synthesized from condensations via Friedländer reaction [14], between intermediate **(2)** and the appropriate di-carbonyl compound as shown in Scheme 2 and Scheme 3 for the formation of compounds 7,8-dihydro-7,7-dimethyl-1,3-dioxolo[4,5-b]acridin-9(6h)-one **(3)** and 6-methyl-[1,3]dioxolo[4,5-g]quinone-7-carboxilate **(4)**. Due to the characteristics of the dicarbonyl compound used, the synthesis of compound **(3)** was performed under acid media. The acid catalysis promotes the protonation of one of the carbonyl groups of the di-carbonyl compound, allowing the formation of a double bond inside the ring and favoring the nucleophile attack to intermediate **(2)**. This way the aldol bond is formed, followed by the imine formation (see mechanism in Figure S1 of Supplementary Materials). Through this reaction, compound **(3)** was obtained as a brown solid with melting point ranging between 176–178 °C, and 68% of yield [17].

The synthesis of compound **(4)** was performed under basic media. In this case the basic catalysis allowed the formation of an efficient nucleophile from the di-carbonyl compound (Scheme 3), favoring the nucleophile attack to compound **(2)** (see proposed mechanism for this reaction in Figure S2 of Supplementary Materials). This reaction produced compound **(4)** as a white solid with melting point around 207–209 °C and 53% of yield [18].

**Scheme 4:**
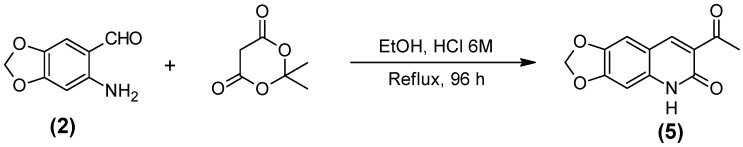
Synthesis of compound (**5**).

The acid 5,6-diidro-6-oxo-1,3-dioxolo [4,6-g] quiniline-7-carboxilic **(5)** was obtained from the reaction between **(2)** and the Meldrum acid, as shown in Scheme 4. For production of **(5)** after aldol condensation and formation of the hydroxyl-imine, the hydrolysis of the Meldrum acid ring with elimination of one molecule of acetone is necessary. A possible mechanism for this reaction is shown in Figure S3 of Supplementary Materials.

**Scheme 5:**
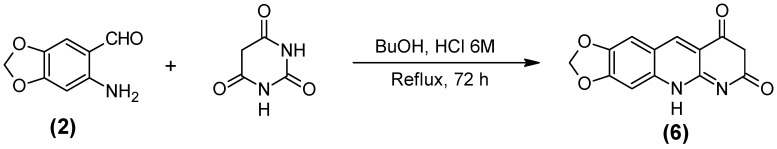
Synthesis of compound (**6**).

Compound **(6)** (1,3-dioxolo [4,5-g] pirimido [4,5-b] quinoline-7,9 (6H,8H)-dione [17]) was synthesized from the reaction between compound **(2)** and pyrimidine-2,4,6 (1H, 3H, 5H)-trione as shown in Scheme 5. Dioxane and ethanol were used as solvents in the early attempt of synthesis of this compound but both solvents led to low yields. The use of the higher boiling point solvent butanol allowed rising the reaction temperature and achieving 62% of yield.

**Scheme 6:**
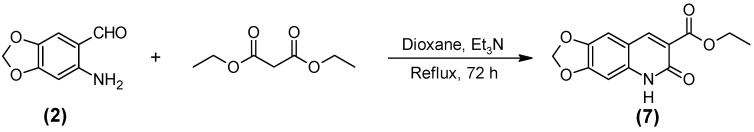
Synthesis of compound (**7**).

Compound **(7)** (ethyl 5,6-dehidro-6-oxo-[1,3] dioxolano [4,5-g] quinoline-7-carboxilate) [19] was synthesized from the reaction between compound **(2)** and diethyl propanedioate, as shown in Scheme 6. The replacement of butanol by dioxane as solvent allowed the obtainment of a sole product. As dioxane is an inert solvent, its use avoided the formation of secondary products due to transterification.

**Scheme 7:**
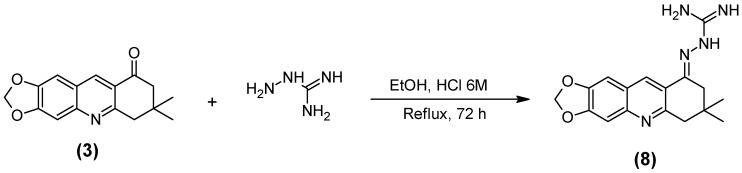
Synthesis of compound (**8**).

Compound **(8)** (guanylhydrazone of 7,7-dimethyl-7,8-dihydro-[1,3]dioxolo[4,5-b]acridin-9 (6h)-one) was synthesized from the reaction between compound **(3)** and hydrazinecarboximidamide, as shown in Scheme 7. This reaction was also performed under acid media because the protonation of the carbonyl oxygen of **(3)** makes the subsequent nucleophile substitution more efficient, allowing the formation of the guanylhydrazone. Compound **(8)** was obtained as a dark yellow solid with melting point around 269–271 °C and 51% of yield [20]. This compound was also found to be never reported before in the literature.

**Figure 2:**
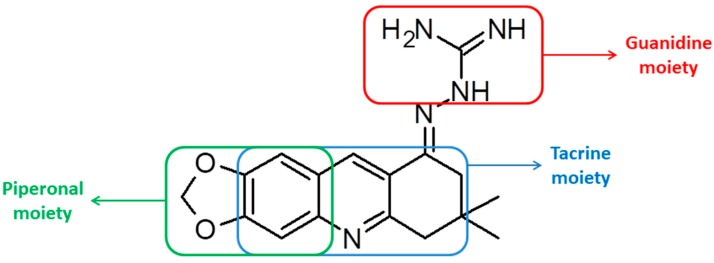
Different chemical classes present in the structure of compound **(8)**.

Compound **(8)** represents the junction of three different chemical classes in the same molecule, where it is possible to find the piperonal, quinoline and guanylhydrazone moieties (see Figure 2). It was expected that these different groups enable interactions with the active site of AChE, like tacrine does, as well as the PAS, due to the positive charges of the guanidine group. It was also expected that the oxole group, from the piperonal moiety, enables H-bond interactions with residues of the active site, increasing the affinity of this compound for the enzyme.

### 2.2. Calculation of Pharmacokinetic and Toxicological Properties

The tridimensional structures of the compounds studied and the values of weight (amu), area (Å^2^), volume (Å^3^), and polar surface area (PSA, Å^2^) were obtained with the software Spartan’08, using Density Functional with M06 and the basis set 6-311++G** [21]. Some pharmacological properties were calculated by the online program Pre ADMET (https://preadmet.bmdrc.kr/adme-prediction/): toxicity, Lipinski’s rule (rule of five) [22,23] and blood–brain barrier (BBB) penetration. The values obtained for compounds **(3)**–**(8)** and tacrine are shown in Table 1.

According to the Lipinski’s rule of five [22,23], the PSA must present values ≤ 140 and ≤ 90 Å^2^, respectively, to provide good oral bioavailability and good penetration into the BBB. Therefore, our results (Table 1) suggest both a good oral bio-availability, in addition to capability of penetrating the BBB and entering the central nervous system (CNS), for all compounds studied. Among them, according to the model proposed by Ma et al. [24], compounds **(3)**, **(4)**, **(6)** and tacrine would be the ones with higher CNS uptake, while compounds **(5)**, **(7)** and **(8)** would show average uptake by the CNS. 

Regarding acute toxicity, (algae) compound **(8)** was the only one presenting a calculated value lower than tacrine’s. This result suggests that this compound can be a suitable lead for the design of less toxic anti-AD drugs. Together with **(3)**, **(4)** and **(7)**, compound **(8)** also showed a negative prediction for rodent carcinogenicity.

### 2.3. Cholinesterase Inhibition Assay through Ellman’s Method

The inhibitory activities of compounds **(3)**–**(8)** were evaluated through the modified spectrophotometric Ellman’s method [25,26] on *Ee*AChE and butyrylcholinesterase from equine serum (EqBChE). Results (Table 2) show that while compound **(4)** is not able to inhibit any of the enzymes, compound **(8)** is a non-selective inhibitor of both enzymes, being twice more potent in inhibiting *Ee*AChE (IC_50_ = 8.50 ± 0.39 μM ) than EqBChE (IC_50_ = 15.16 ± 0.75 μM). The other compounds (**(3)**, and **(5)**–**(7)**) selectively inhibited *Ee*AChE. Tacrine, a non-selective inhibitor of both enzymes, with major selectivity towards BChE, instead of AChE [27], was the reference compound. 

The compounds synthesized in this work were designed with a dioxole ring because according to the literature this ring is important for AChE inhibition, due to the capacity of accepting H-bonds of the 3,4-dioxole moiety [12,28,29]. Our compounds also present two aromatic rings in the middle of the molecule. This confers rigidity and planarity, allowing π–π interactions with the aromatic residues of the AChE gorge [20,30,31]. Comparing compounds **(3)** and **(8)** it is possible to see that they differ only by the substitution of a carbonyl group in compound **(3)** by an amino-guanidine in compound **(8)**. This structural change conferred to **(8)** an inhibitory activity over *Ee*AChE six times higher than **(3)**, besides making this compound also active towards EqBChE.

According Sugimoto et al. [30], carbonyl groups conjugated to aromatic rings are important for inhibition of AChE. However, Ferreira Neto et al. [25] showed that the presence of a guanyl-hydrazone allows interactions with the catalytic anionic site (CAS) of AChE, improving the activity, as also shown here.

### 2.4. Kinetic Study of Compound (8) by NMR

As compound **(8)** presented the lowest IC_50_ value in Ellman’s test, it was selected for an additional kinetic study by NMR [32] in order to verify the percentage of inhibition when compared to the reference drug (tacrine). The enzyme selected for this study was *Ee*AChE because it displays an identical active site and a very similar activity to the human enzyme [33,34].

**Figure 3:**
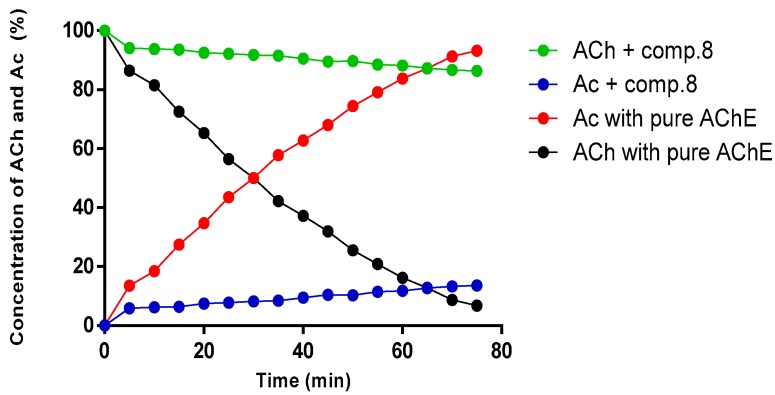
AChE inhibition by compound **(8)** using the NMR test.

NMR is a good method for the biological evaluation of AChE inhibitors. Recent published works have indicated results consistent with Ellman’s method [25,32,33,34,35,36]. In our study we used the NMR method [29] to determine the concentrations of the substrate (ACh) and the product acetate (Ac), which are obtained from direct integration of the corresponding absorption peaks for methyl groups (2.24 ppm for ACh and 2.16 ppm for Ac) [32,35]. The comparison between the integration of the Ac peak in pure AChE (no inhibitor) and in the presence of the inhibitor allowed the determination of the percentages of inhibition shown in the plot of Figure 3.

Results showed that compound **(8)** presented 73.90 ± 6.26 of AChE inhibition *versus* 93.50 ± 5.42 of tacrine. This corroborates recent studies [13,25,36] showing that the presence of an electrophilic region in the molecule (the guanylhydrazone moiety) may favor interactions with the enzyme and thus allow its inhibition to be more effective. This result reaffirms the importance of this class as promising for the inhibition of AChE and suggests that similar derivatives should be developed as prototypes for the design of more efficient drugs.

### 2.5. Docking Studies 

#### 2.5.1. Docking Studies on *Ee*AChE

The root mean square deviation (RMSD) values obtained for tacrine inside *Ee*AChE and EqBChE were 0.28 and 0.30 respectively. These values are enough to validate our docking protocol because, according to the literature [37], RMSD values < 2.50 Å are considered acceptable. The re-docking poses obtained for tacrine inside *Ee*AChE and EqBChE are shown in Figure S4 of Supplementary Materials.

**Figure 4:**
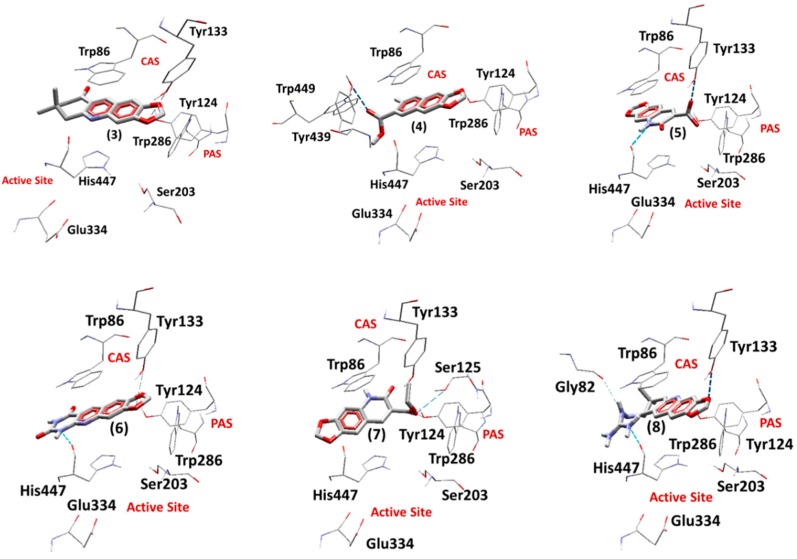
Best docking poses of the ligands inside *Ee*AChE.

Table 3 presents the values of energy for the enzyme–ligand interactions and H-bonds, as well as the corresponding interacting residues, for the best poses of each ligand inside *Ee*AChE, obtained from the docking studies (Figure 4). As can be seen, compounds **(3)**–**(8)** presented negative values of intermolecular energy inside *Ee*AChE. This suggests that they present stabilization in the region between the CAS and PAS. Residues Tyr124 (PAS residue), Tyr133 (CAS residue) and His447 (which is a residue of the catalytic triad) contributed to the stabilization of the ligands, with compound **(8)** as the one presenting the lowest intermolecular energy. This is in accordance with the experimental results pointing to this compound as the best AChE inhibitor among the studied compounds.

#### 2.5.2. Docking Studies on EqBChE

**Figure 5:**
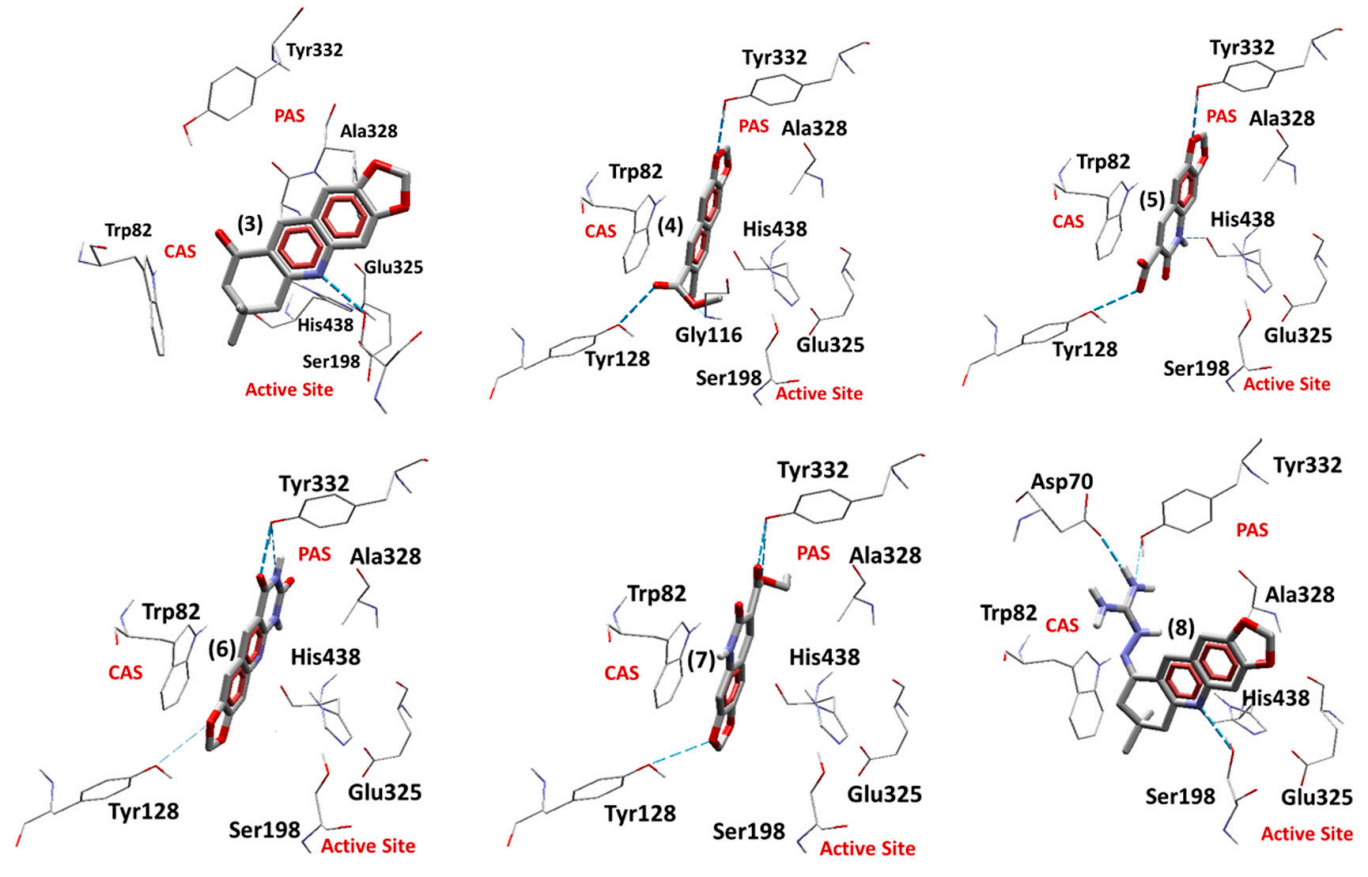
Best docking poses of the ligands inside EqBChE.

Table 4 presents the values of energy for the enzyme-ligand interactions and H-bonds, as well as the corresponding interacting residues, for the best poses of each ligand inside EqBChE, obtained from the docking studies (Figure 5). All the compounds presented negative values of intermolecular energy, suggesting that they present stabilization inside EqBChE. Compound **(8)** presented the lowest intermolecular energy, pointing to a better stabilization than other compounds. The residues Asp70, Tyr332 (PAS residues), Tyr128 (CAS residue) and His438 (which is a residue of catalytic triad) contributed to the stabilization of the ligands. Regarding the experimental results, it is important to notice that, besides tacrine, **(8)** was the only compound that was able to also inhibit EqBChE.

## 3. Materials and Methods

### 3.1. Chemistry

Solvents (dioxane, ethyl alcohol, methanol, chloroform), used with further purification, were purchased from VETEC, and reagents were purchased from Sigma-Aldrich. Reactions were monitored by TLC using DCAlufolien Kieselgel 60 F254 (Merck, Kenilworth, NJ, USA).

### 3.2. Synthesis of The Compounds

#### 3.2.1. 6-Nitro-1,3-benzodioxole-5-carboxaldehide [6-nitropiperonal, (1)]

The synthesis was done following the procedure described in literature [14,15] with some modifications to improve the yield. Firstly, 20.0 g of piperonal powdered to a fine dark brown powder was added to 100 mL of HNO_3_ 65% PA, over an ice bath, under constant stirring. After 3 h of reaction the light yellow precipitate that formed was filtered and washed with cold water till total removal of the HNO_3_ [11,12]. Physical aspect: light yellow solid, yield: 91%. NMR ^1^H (DMSO-d_6_, 600 MHz): δ/ppm 10.08 (1H,s); 7.74 (1H,s); 7.32 (1H,s); 6.33 (2H,s); NMR ^13^C (DMSO-d_6_, 125 MHz): δ/ppm 188.18; 151.89; 151.31; 145.67; 127.47; 107.04; 104.99; 104.40.

#### 3.2.2. 6-Amino-1,3-benzodioxole-5-carboxaldehide [(6-aminopiperonal, (2)]

Firstly, 1.00 g of compound **(1)** was first solubilized in 50.0 mL of boiling ethanol 50%. Next, a solution of 10 g of ferrous sulfate heptahydrate, in 50.0 mL of distilled water, was added to the flask and left under heating for 1 min. After this time, 1.00 mL aliquots of NH_4_OH concentrated were added at intervals of 30 s till achieving 13.0 mL, and left at 100 °C for 10 min. The yellow solution obtained after filtration produced a dark yellow precipitate after cooling for some hours in an ice bath [16,38]. This precipitate was recrystallized in distilled water, producing dark yellow needles. Physical aspect: dark yellow solid; yield: 77%, NMR ^1^H (DMSO-d_6_, 600 MHz): δ/ppm 9.54 (1H,s); 7.24 (2H,bs); 6.98 (1H,s); 6.31(1H,s); 5.96 (2H,s); NMR ^13^C (DMSO-d6, 125 MHz): δ/ppm 190.49; 153.55; 150.12; 138.10; 110.95; 110.66; 101.26; 95.18.

#### 3.2.3. 7,8-Dihydro-7,7-dimethyl-1,3-dioxolo[4,5-b]acridin-9(6h)-one (3)

Firstly, 1 mmol of dimedone (0.140 g), 20.0 mL of dioxane and 1 mmol of compound **(2)** (0.165 g) solubilized in 20.0 mL of dioxane were mixed in a tritubed flask and left under reflux for 72 h [17]. The product was purified through chromatographic column. Physical aspect: brown solid; yield: 68%; NMR ^1^H (DMSO-d_6_, 600 MHz): δ/ppm 8.56 (1H,s); 7.44 (1H,s); 7.25 (1H,s); 6.17 (2H,s); 2.99 (2H,s); 2.50 (2H,s); 0.96 (6H,s); NMR ^13^C (DMSO-d_6_, 125 MHz): δ/ppm 197.84; 159.23; 153.27; 148.91; 148.01; 134.32; 123.70; 104.76; 104.47; 102,86; 52.04; 46.21; 32.89; 28.26.

#### 3.2.4. Methyl 6-Methyl-[1,3]dioxolo[4,5-g]quinone-7-carboxilate (4)

Firstly, 1 mmol of compound **(2)** (0.165 g) was solubilized in 10.0 mL of methanol and mixed with 1 mmol of ethyl acetoacetate and a catalytic amount of triethylamine. The solution was maintained under stirring and reflux for 120 h [18]. The white precipitate obtained after this time was filtered, washed and dried. Physical aspect: white solid; melting point: 207-209 °C; yield: 53%. NMR ^1^H (DMSO-d_6_, 600 MHz): δ/ppm 8.65 (1H,s); 7.45 (1H,s); 7.30 (1H,s); 6.23 (2H,s); 3.88 (3H,s); 2.78 (3H,s). NMR ^13^C (DMSO-d_6_, 125 MHz): δ/ppm 166.49; 155.40; 152.53; 147.63; 146.93; 138.06; 122.25; 121.04; 104.30; 103.33; 102.37; 52.18; 24.79.

#### 3.2.5. Acid 5,6-Diidro-6-oxo-1,3-dioxolo[4,6g] quiniline-7-carboxilic (5)

Firstly, 1 mmol of compound **(2)** (0.165 g) were added to a tritubed flask and solubilized under heating in 20.0 mL of dioxane. Then, 1 mmol of meldrum acid (0.144 g) solubilized in 20.0 mL of dioxane was added and the resulting solution kept under reflux. Next, 16 drops of HCl 6 M were added to this solution to promote an acid catalysis. After 16 h the formation of the product was observed by TLC. Physical aspect: brown solid; melting point > 300 °C; yield: 56 %. NMR ^1^H (DMSO-d_6_, 600 MHz): δ/ppm 14.85 (1H,s); 13.14 (1H,bs); 8.81 (1H,s); 7.51 (1H,s); 6.98 (1H,s); 6.21 (2H,s). NMR ^13^C (DMSO-d_6_, 125 MHz): δ/ppm 164.99; 163.73; 153.60; 145.37; 145.10; 138.01; 114.44; 113.94; 106.23; 102.84; 95.41.

#### 3.2.6. 1,3-Dioxolo [4,5-g] pirimido [4,5-b] quinoline-7,9 (6H,8H)-dione (6)

Firstly, 20.0 mL of butanol and 1 mmol of compound **(2)** (0.165 g) were added to a tritubed flask. Then, a solution containing 1 mmol of barbituric acid (0.128 g) in 20.0 mL of buthanol was added to this solution. Next, 15 drops of HCl 6M were added, and the resulting solution kept under reflux for 4 h. The rose-colored precipitate formed was filtered and dried. Physical aspect: pink solid; melting point > 300 °C; yield: 62%; NMR ^1^H (DMSO-d_6_, 600 MHz): δ/ppm 11.55 (1H,bs); 11.39 (1H,s); 8.74 (1H,s); 7.50 (1H,s); 7.19 (1H,s); 6.24 (2H,s); NMR ^13^C (DMSO-d_6_, 125 MHz): δ/ppm 162.25; 153.53; 150.49; 148.94; 148.57; 146.62; 136.61; 121.18; 108.28; 103.88; 103.04; 102.42.

#### 3.2.7. Ethyl 5,6-Dehidro-6-oxo-[1,3] dioxolano [4,5-g] quinoline-7-carboxilate (7)

Firstly, 1 mmol of compound **(2)** (0.165 g) solubilized in 10.0 mL of dioxane was added to 1 mmol of diethyl malonate in 10.0 mL of dioxane in a tritubed flask. After 72 h of reflux, the light green precipitated obtained was filtered, washed and dried. Physical aspect: light green solid; melting point; 241–243 °C; yield: 71 %. NMR ^1^H (DMSO-d_6_, 600 MHz): δ/ppm 11.92 (1H,s); 8.39 (1H,s); 7.31 (1H,s); 6.80 (1H,s); 6.13 (2H,s); 4.24 (2H,q, 7.0Hz); 1.28 (3H,t, 7.0Hz). NMR ^13^C (DMSO-d_6_, 125 MHz): δ/ppm 164.45; 158.34; 152.30; 143.82; 143.61; 138.38; 119.09; 112.02; 105.98; 102.19; 94.55; 60.32; 14.18.

#### 3.2.8. Guanylhydrazone of 7,7-Dimethyl-7,8-dihydro-[1,3]dioxolo[4,5-b]acridin-9(6h)-one (8)

Firstly, 1 mmol of aminoguanidine chloridrate (0.111 g), 10.0 mL of ethanol, and 10 drops of HCl 6 M were mixed in a tritubed flask and kept under reflux for 10 min. Then, 1 mmol of compound **(3)** (0.269 g) and 10 mL of ethanol were added, and the resulting solution kept under reflux for 72 h [20]. The dark yellow precipitate obtained was filtered, washed and dried. Physical aspect: dark yellow solid; melting point: 269–271 °C; yield: 51 %. NMR ^1^H (DMSO-d_6_, 500 MHz): δ/ppm 11.63 (1H,s); 9.74 (1H,s); 8.03 (4H,bs); 7.68 (1H,s); 7.45 (1H,s); 6.39 (2H,s); 3.18 (2H,s); 2.73 (2H,s); 1.06 (6H,s). NMR ^13^C (DMSO-d6, 125 MHz): δ/ppm 156.08; 154.42; 152.58; 149.27; 146.85; 139.51; 137.40; 124.92; 123.84; 103.82; 103.39; 98.28; 56.02; 38.50; 30.49; 27.87. IV (KBr, cm^−1^): 3318; 2963; 2700; 1682; 1597; 1466; 1366; 1265; 1142; 1034; 933.

### 3.3. NMR Analysis

The NMR analysis of compounds was executed using a Varian 600 MHz (Premium compact TM) using tubes of 5 mm and DMSO-d_6_ as solvent. All spectra were executed at 25 °C and using 20 mg of each sample. The NMR spectra produced were ^1^H, ^13^C, APT, Gated Decoupling ^13^C, gHSQC, gHMBC and ROESY-1D.

### 3.4. Structure Optimization and Calculation of Pharmacokinetic and Toxicological Properties

As before [32], the structures of the new compounds were built and optimized, using the density functional theory (DFT) with M06 and 6-311++G** of Spartan08^®^ [21]. In addition, the values of Weight (amu), Polar Surface Area (PSA, Å^2^), Area (Å^2^) and their Volume (Å^3^) were obtained. Some pharmacological properties were calculated through the online program Pre ADMET (https://preadmet.bmdrc.kr/adme-prediction/): toxicity, Lipinski’s rule (rule of five) and blood–brain barrier penetration (BBB). These predictions are important for the characterization of these compounds and to evaluate if they have the capacity as pharmacological agents.

### 3.5. Cholinesterase Inhibition Assay by Ellman’s Method

In order to determine the anticholinesterase activity, we used Ellman’s method [26] modified for a 96-wells plate [39]. The velocities of substrate hydrolysis by AChE and BChE as function of sample concentration were evaluated for *Ee*AChE and EqBChE. AChE, BChE and Ellman’s reagent (DTNB) were prepared in phosphate buffer (100 mM, pH 7.4). Acetylthiocholine iodide (ATCI), and butyrylthiocholine iodide (BTCI) were prepared in distilled water. Stock samples (100 mM) were prepared in dimethyl sulphoxide (DMSO) and appropriately diluted in distilled water to the desired concentrations immediately before use. All solutions were kept on ice during the experiment. All experiments were performed at 37 ± 1 °C.

All experimental wells received *Ee*AChE (0.01 U/mL) or EqBChE (0.05 U/mL), DTNB (0.25 mM), and phosphate buffer (control—enzyme activity) or sample solutions (1 to 100 µM). The mixture was incubated for 10 min. Then, ATCI (0.5 mM) or BTCI (1.0 mM) was added to all wells and the plate was read immediately for 5 min in a spectrophotometer (Spectramax 340PC, Molecular Device^®^). The spontaneous and sample (100 µM) induced hydrolysis of the substrate were evaluated by replacing enzyme for buffer. No sample-induced hydrolysis was observed for the compounds studied. The solvent (DMSO) was evaluated at the highest concentration (0.2%) used in the experiment. All concentrations refer to final concentrations. The samples were tested in at least 5 concentrations. The enzyme activity (absorbance/min) in the samples was determined by comparison with the control (mixture without sample) and expressed as the change in the optic deviation at 412 nm. The values of absorbance/min were calculated by the software Softmax Pro 6.4^®^. Inhibition values were calculated through non-linear regression with the software GraphPadPrism 5 (GraphPad software^®^, San Diego, CA, USA). For each sample, results correspond to average ± standard deviation of three experiments, each one being performed in triplicate. *Ee*AChE (EC.3.1.1.7), EqBChE (EC3.1.1.8), ATCI, BTCI and DTNB were purchased from Sigma-Aldrich (São Paulo, Brazil).

### 3.6. AChE Inhibition Assay by NMR Method

All NMR analyses were performed using an NMR method formerly used in our research group [32] on a Varian Premium COMPACT TM 600 MHz spectrometer using a 5-mm probe at 25 °C. Two microliters of *Ee*AChE 10 μM in phosphate buffer (100% D_2_O, pH 7.4) in the presence of 1% bovine serum albumin was used. This enzyme solution was mixed with 30 μL of ACh (100 mM in D_2_O), and then it was diluted to 600 μL using phosphate buffer (100 mM, 100% D_2_O, pH 7.4) in the NMR tube (final concentration = 5 mM for ACh and 33 nM for the enzyme). This sample was immediately inserted in the magnet for locking and shimming, allowing for the observation of the first ^1^H spectra exactly 5 min after the addition of ACh. The next ^1^H spectra were acquired at every 5 min with a single scan over 75 min. For testing the new compound, the same procedure was performed, including the addition of 5 μL of the potential *Ee*AChE inhibitor (final concentration of the inhibitor in the NMR tube = 10 mM) before the addition of ACh. The concentrations of ACh and Ac were determined by the integration of the methyl signals (ACh at 2.24 ppm and Ac at 2.16 ppm). The values generated from the integration of the signals of ACh (consumed during the reaction) and Ac (produced during the reaction) are compared between each other and to the values of Ac produced in the presence and in the absence of the inhibitor. This enables the calculation of the percentages of inhibition, as described before [32]. All analyses were performed in triplicate.

### 3.7. Docking Studies

The protonation states of the ligands at pH = 7.4 were obtained using the server Chemicalize web-based resource supported by ChemAxon Ltd. at https://chemicalize.com/welcome [40]. This server predicts aqueous ionization constants (pKa) of organic molecules based mainly on empirically calculated partial charges and parameterized H-bonds. The tridimensional structures of the most abundant chemical species of each ligand as calculated by the Chemicalize server [40] were constructed with the software PC Spartan Pro^®^ [21] and had their partial atomic charges calculated by the RM1 (Recife Model 1) semi-empirical method [41].

The model of EeAChE inhibited by tacrine was constructed from the crystallographic structure of AChE from *Torpedo californica* (*Tc*AChE) inhibited by tacrine available in the RCSB PDB Protein Data Bank (http://www.rcsb.org/pdb/home/home.do), under the code 1ACJ, and the crystallographic structure of *Ee*AChE, available in PDB under the code 1C2B. These two structures were aligned using the software spdbviewer [42] and the coordinates of tacrine were copied from the *Tc*AChE to the *Ee*AChE file. The model of EqBChE inhibited by tacrine was constructed from the crystallographic structure of BChE from *Homo sapiens sapiens* (*Hss*BChE) complexed with tacrine in the active site, available in the RCSB PDB Protein Data Bank (http://www.rcsb.org/pdb/home/home.do), under the code 4BDS. EqBChE and *Hss*BChE present 90% of identity and the active site 100% conserved. The Fasta sequence of EqBChE was aligned to *Hss*AChE and further submitted to the Swiss Model server [43] for construction of the model. Then, the structures of template (*Hss*BChE) and model were aligned using the software spdbviewer [42] and the coordinates of tacrine were copied from *Hss*BChE to EqBChE.

The models *Ee*AChE/tacrine and EqBChE/tacrine obtained were validated using the server PDBSum (www.ebi.ac.uk/pdbsum/), where Ramachandran plot and G-factors such as dihedral angles and covalent forces related to bond angles and length were analyzed. After validation, the ligands were docked inside each model using the software Molegro Virtual Docker (MVD)^®^ [44]. The binding site inside *Ee*AChE was restricted into a sphere with a radius of 10 Å, centered on the coordinates x = 8.95, y = 68.42 and z = 64.80, while for EqBChE it was centered on the coordinates x = 133.17; y = 116.22 and z = 40.98. For both models the residues 6 Å closest to tacrine were considered flexible. The best conformation of each compound was selected from a set of 300 results (poses) obtained for each ligand, according to the binding energies and hydrogen bonds (H-bond) interactions criteria. The docking protocol used was validated through re-docking of tacrine inside *Ee*AChE and EqBChE.

## 4. Conclusions

The reactions among compound **(2)** and the carbonyl compounds were confirmed to be very efficient for the synthesis of quinolines. The synthesis of the guanylhydrazone **(8)** through the reaction between a quinoline and chloridrate of amino guanidine also showed to be efficient, presenting good yields. 

The compounds derived from 1,3-benzodioxole-5-carboxaldheide synthesized were evaluated as inhibitors of AChE. The results obtained were already expected because quinolines and guanyl hydrazones are molecules with a wide range of applications in medicinal chemistry and bear structural characteristics essential to enable interactions with the active site of AChE. The presence of the aromatic rings resembling the base structure of tacrine in the quinolines synthesized enables π–π with the amino acids present in the active site, while the guanyl hydrazone was capable of interacting with the CAS of AChE through its guanidine group. Compounds **(6)** and **(8)** presented promising results as AChE inhibitors with results for compound **(8)** being similar to the known AChE inhibitor rivastigmine. To the best of our knowledge, the synthesis of this compound was reported here for the first time in the literature.

Comparison between structures of compounds **(3)** and **(8)** allowed to observe that the presence of a guanylhydrazone contributes to enhancing the inhibitory capacity of this kind of compound.

## Figures and Tables

**Table 1:** Values of weight (amu), area (Å^2^), volume (Å^3^), polar surface area (PSA, Å^2^), acute toxicity, Lipinski’s rule and blood–brain barrier (BBB) calculated for compounds **(3)**–**(8)**.

Comp	Weight (amu)	Area (Å^2^)	Volume (Å^3^)	PSA (Å^2^)	Acute Toxicity (algae)	Carcinog. (mouse)	Meet the Lipinski’s Rule?	BBB Penetration
**3**	269.30	275.40	266.12	38.55	0.05	Negative	Yes	2.69
**4**	245.23	250.08	233.59	45.63	0.09	Negative	Yes	1.37
**5**	233.18	216.47	202.36	70.70	0.15	Positive	Yes	0.29
**6**	257.21	235.21	221.47	75.45	0.19	Positive	Yes	0.39
**7**	261.23	260.62	242.00	63.40	0.12	Negative	Yes	0.04
**8**	325.37	334.47	317.07	89.56	0.03	Negative	Yes	0.11
**Tacrine**	198.27	225.26	213.94	30.24	0.05	Positive	Yes	0.86

**Table 2:** IC_50_ values, in μM, of compounds **(3)**–**(8)** compared to tacrine.

Compound	*Ee*AChE	EqBChE
**(3)**	51.51 ± 2.47	-
**(4)**	-	-
**(5)**	14.15 ± 1.15	-
**(6)**	32.06 ± 3.53	-
**(7)**	62.76 ± 2.02	-
**(8)**	8.50 ± 0.39	15.16 ± 0.75
Tacrine	0.0414 ± 0.0002	0.0045 ± 0.0005

**Table 3:** Docking results inside AChE from *Electrophorus electricus* (*Ee*AChE).

Ligand	E_inter_ (Kcal.mol^−1^)	E_H-bond_ (Kcal.mol^−1^)	H-bond Interactions	RMSD (Å)
**Redocking Tacrine**	−132.39	−0.20	His447	0.28
**(3)**	−132.69	−0.13	Tyr133	-
**(4)**	−135.51	−0.18	Trp439, Tyr449	-
**(5)**	−140.88	−0.22	Tyr133	-
**(6)**	−145.33	−1.76	Tyr133, His447	-
**(7)**	−119.49	−0.46	Tyr124, Ser125	-
**(8)**	−155.88	−2.35	Tyr133, His447	-

**Table 4:** Docking results on butyrylcholinesterase from equine serum (EqBChE).

Ligand	E_inter_ (Kcal.mol^−1^)	E_H-bond_ (Kcal.mol^−1^)	H-bond Interactions	RMSD (Å)
**Redocking Tacrine**	−133.63	−2.08	His438	0.30
**(3)**	−153.23	−2.50	Ser198	-
**(4)**	−152.11	−5.27	Gly116, Tyr128, Tyr332	-
**(5)**	−155.08	−5.73	Tyr128, Tyr332, His438	-
**(6)**	−151.08	−3.96	Tyr128, Tyr332	-
**(7)**	−158.06	−4.26	Tyr128, Tyr332	-
**(8)**	−196.99	−5.43	Asp70, Ser198, Tyr332	-

## Supplementary Materials

Supplementary materials can be found at https://www.mdpi.com/1422-0067/20/16/3944/s1.

## Abbreviations

ACh	Acetylcholine
AChE	Acetylcholinesterase
AD	Alzheimer Disease
ATCI	Acetylthiocholine Iodide
BBB	Blood Brain Barrier
BChE	Butyrilcholinesterase
BTCI	Butyrylthiocholine Iodide
CAS	Catalytic Anionic Site
CNS	Central Nervous System
DFT	Density Functional Theory
DMSO	Dimethylsulfoxide
DMSO-d_6_	Deuterated DMSO
DTNB	5,5-dithio-bis-(2-nitrobenzoic acid)
*Ee*AChE	*Electrophorus electricus* AChE
EqBChE	Equine serum BChE
IR	Infrared
MHz	Megahertz
NMR	Nuclear Magnetic Ressonance
PAS	Peripheral Anionic Site
PDB	Protein Data Bank
PSA	Polar Surface Area
RMSD	Root Mean Square Deviation
TLC	Thin Layer Chromatography
