# Supplementary material for: Synthesis of New Quinoline-Piperonal Hybrids as Potential Drugs against Alzheimer’s Disease
Source: Int J Mol Sci. 2019 Aug 14;20(16):3944. doi: 10.3390/ijms20163944 (PMC6720848; doi:10.3390/ijms20163944)
Supplement: Supplementary file 1 [file ijms-20-03944-s001.pdf]

International Journal of *Molecular Sciences*

MDPI

Article

Synthesis of new quinolone-piperonal hybrids as potential drugs against Alzheimer disease

Juliana de Oliveira C. Brum,[1,*] Denise Cristian F. Neto,[1] Joyce Sobreiro F. D. de Almeida,[2] Josélia Alencar Lima,[2,3] Kamil Kuca,[4] Tanos Celmar C. França,[2,4] José D. Figueroa-Villar[1,*]

[1] Medicinal Chemistry Group, Department of Chemistry, Military Institute of Engineering, Praça General Tibúrcio 80, 22290-270, Rio de Janeiro, RJ, Brazil; juliana_brum2005@yahoo.com.br (J.O.C.B.); denisecristian@gmail.com (D.C.F.N.); jdfv2009@gmail.com (J.D.F.V.).

[2] Laboratory of Molecular Modeling Applied to the Chemical and Biological Defense (LMCBD), Military Institute of Engineering, Praça General Tibúrcio 80, 22290-270, Rio de Janeiro, RJ, Brazil; joycesfdalmeida@gmail.com (J.S.F.D.A.); aljoselia@gmail.com (J.A.L.); tanos@ime.eb.br (T.C.C.F.).

[3] Graduate Program in Adult Health, Federal University of Maranhão, Avenida dos Portugueses, 1966. Vila Bacanga, 65080-805, São Luís, MA, Brazil; aljoselia@gmail.com (J.A.L.).

[4] Department of Chemistry, Faculty of Science, University of Hradec Kralove, Rokitanskeho 62, 50003 Hradec Kralové, Czech Republic; kamil.kuca@uhk.cz (K.K.); tanos@ime.eb.br (T.C.C.F.).

* Correspondence: juliana_brum2005@yahoo.com.br (J.O.C.B.); jdfv2009@gmail.com (J.D.F.V.).

Received: date; Accepted: date; Published: date

Supplementary material

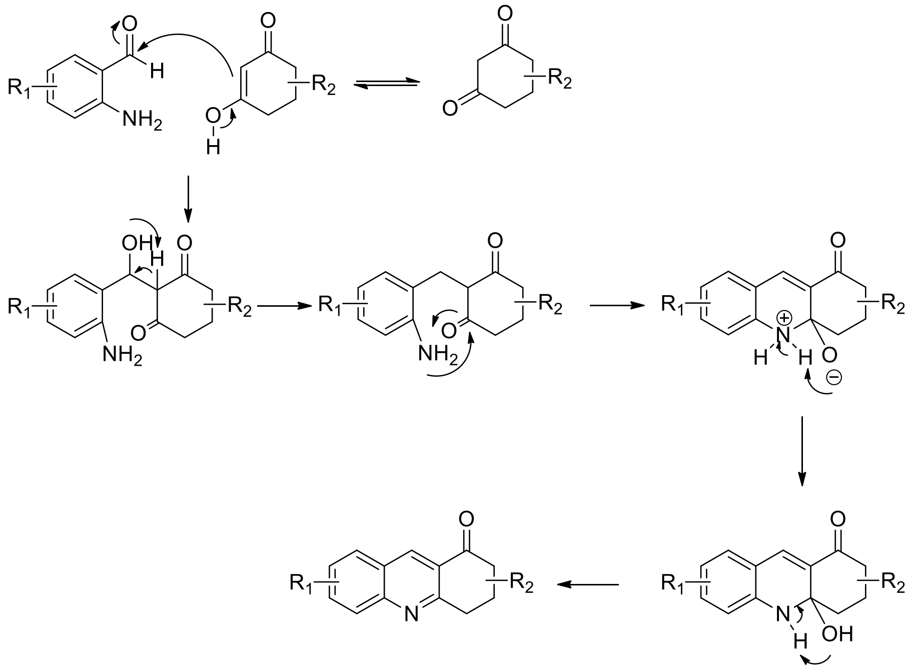

Figure S1. General mechanism for quinolone synthesis through the Friedländer reaction [14].

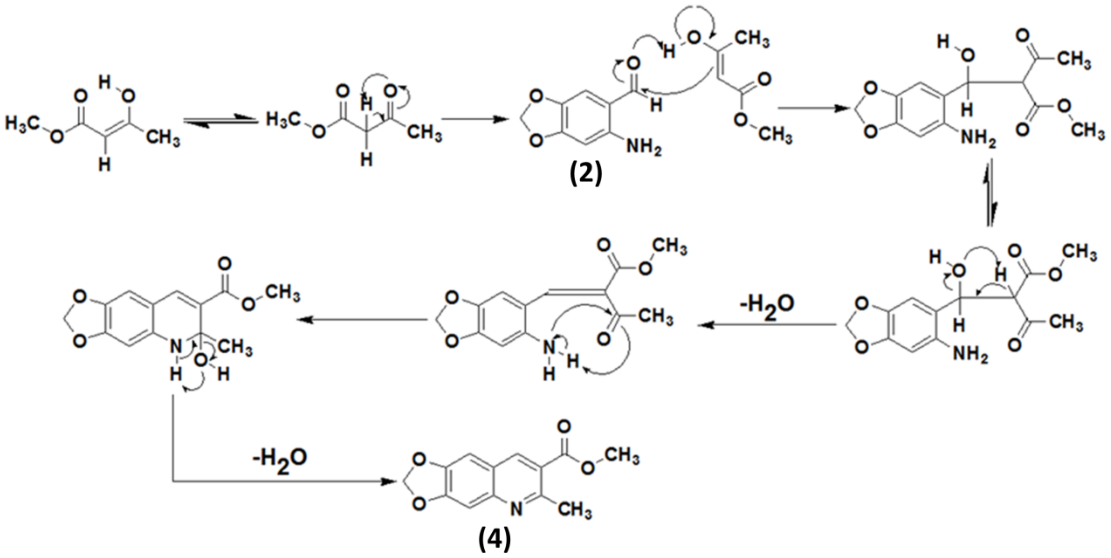

Figure S2. Proposed mechanism for the synthesis of compound **(4)**.

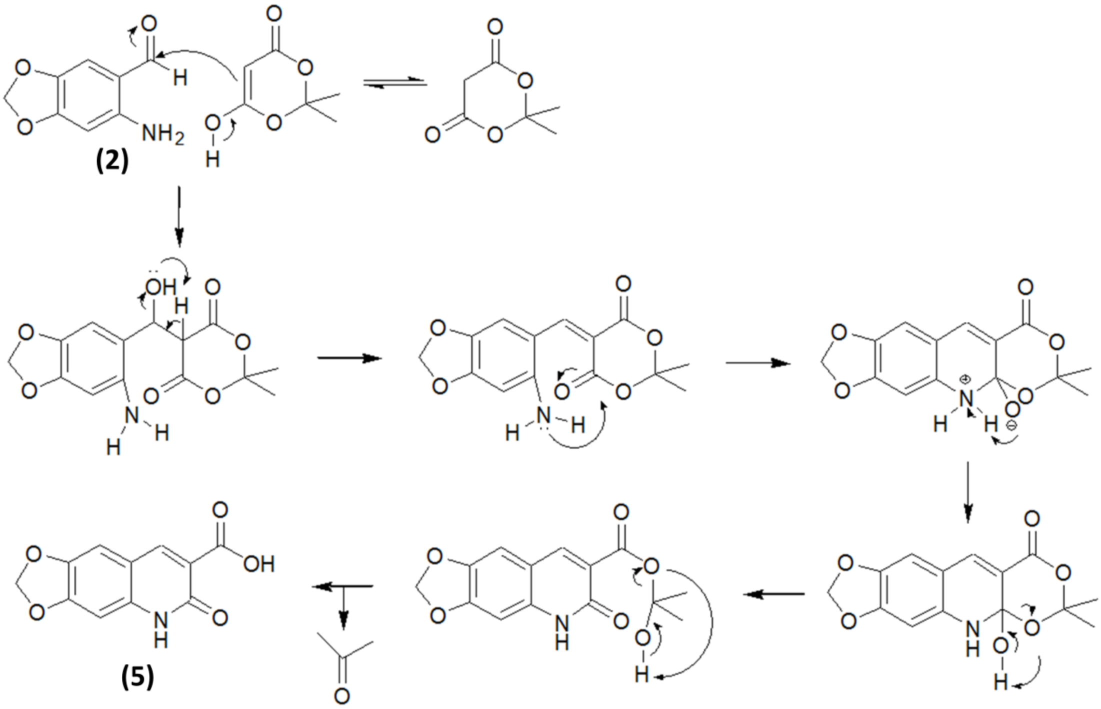

Figure S3. Proposed mechanism for the synthesis of compound **(5)**.

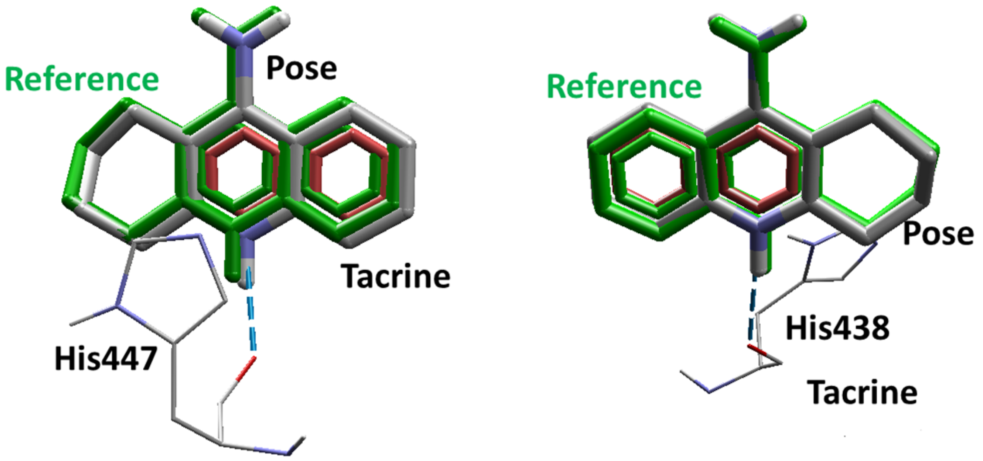

Figure S4. Best re-docknig poses obtained for tacrine inside *Ee*AChE (left) and EqBChE (right). Reference structures are shown in green.

© 2019 by the authors. Submitted for possible open access publication under the terms and conditions of the Creative Commons Attribution (CC BY) license (http://creativecommons.org/licenses/by/4.0/).
